# Using Machine Learning Approaches to Predict Target Gene Expression in Rice T-DNA Insertional Mutants

**DOI:** 10.3389/fgene.2021.798107

**Published:** 2021-12-17

**Authors:** Ching-Hsuan Chien, Lan-Ying Huang, Shuen-Fang Lo, Liang-Jwu Chen, Chi-Chou Liao, Jia-Jyun Chen, Yen-Wei Chu

**Affiliations:** ^1^ Ph.D. Program in Medical Biotechnology, National Chung Hsing University, Taichung, Taiwan; ^2^ Biotechnology Center, National Chung Hsing University, Taichung, Taiwan; ^3^ Institute of Molecular Biology, National Chung Hsing University, Taichung, Taiwan; ^4^ Advanced Plant Biotechnology Center National Chung Hsing University, Taichung, Taiwan; ^5^ Institute of Genomics and Bioinformatics, National Chung Hsing University, Taichung, Taiwan; ^6^ Agricultural Biotechnology Center, National Chung Hsing University, Taichung, Taiwan; ^7^ Ph.D. Program in Translational Medicine, National Chung Hsing University, Taichung, Taiwan; ^8^ Rong Hsing Research Center for Translational Medicine, National Chung Hsing University, Taichung, Taiwan

**Keywords:** rice, CaMV 35S enhancer, T-DNA activation tagging, gene expression, machine learning

## Abstract

To change the expression of the flanking genes by inserting T-DNA into the genome is commonly used in rice functional gene research. However, whether the expression of a gene of interest is enhanced must be validated experimentally. Consequently, to improve the efficiency of screening activated genes, we established a model to predict gene expression in T-DNA mutants through machine learning methods. We gathered experimental datasets consisting of gene expression data in T-DNA mutants and captured the PROMOTER and MIDDLE sequences for encoding. In first-layer models, support vector machine (SVM) models were constructed with nine features consisting of information about biological function and local and global sequences. Feature encoding based on the PROMOTER sequence was weighted by logistic regression. The second-layer models integrated 16 first-layer models with minimum redundancy maximum relevance (mRMR) feature selection and the LADTree algorithm, which were selected from nine feature selection methods and 65 classified methods, respectively. The accuracy of the final two-layer machine learning model, referred to as TIMgo, was 99.3% based on fivefold cross-validation, and 85.6% based on independent testing. We discovered that the information within the local sequence had a greater contribution than the global sequence with respect to classification. TIMgo had a good predictive ability for target genes within 20 kb from the 35S enhancer. Based on the analysis of significant sequences, the G-box regulatory sequence may also play an important role in the activation mechanism of the 35S enhancer.

## 1 Introduction

Rice is one of the most important models of monocotyledon plants for the analysis of plant gene function. Rice is one of three major food crops throughout the world, and it is the staple food of more than half of the world’s population. Rice production has doubled in the past 30 years, although the supply of rice is expected to gradually become insufficient with the rapid increase in the world population, climate change, and a shortage of water (Ray et al., 2013). It will not be easy to increase food production to the necessary levels. In 2004, the International Rice Genome Sequencing Project (IRGSP) completed the sequencing of the rice genome (IRGSP, 2005). The ultimate goal of genome analysis is to realize the structure and function of each gene within an organism. To further confirm the function of and metabolic pathways related to each gene in rice, scientists have focused their efforts on analyzing the rice genome and are committed to promoting rice genome annotation to move rice research into the post-genome era.

T-DNA insertion activation-tagging technology is widely used in the analysis of the function of rice genes (Jeong et al., 2002; Yang et al., 2013). This method results in the construction of four tandem cauliflower mosaic virus (CaMV) 35S enhancers on a T-DNA plasmid; when this T-DNA is inserted into the rice genome, it activates genes that flank the T-DNA insertion site (Hsing et al., 2007). The CaMV 35S enhancer can activate gene expression in dicots and monocots and is widely used in T-DNA transformation. Gene expression gradually increases with the number of 35S enhancers on T-DNA, which led to the incorporation of four tandem repeat CaMV 35S enhancers for enhanced gene expression with this approach (Odell et al., 1985; Fang et al., 1989; Kardailsky et al., 1999; Weigel et al., 2000; Huang et al., 2001; Ichikawa et al., 2003). Agrobacterium-mediated T-DNA transformation tends to insert one copy of T-DNA, an average of 1.4 loci of T-DNA inserts in transgenic plants (Jeon et al., 2000), reducing the complexity of rice gene research. T-DNA inserted into the rice genome with a 35S enhancer resulted in two states:

(1) Gene knockdown: when T-DNA is inserted into the coding region or promoter of a gene, it is likely to destroy the structure of the gene, resulting in reduced function or loss of function of the gene.

(2) Activation tagging: T-DNA might enhance the activity of genes that flank the T-DNA insertion site through the effect of the 35S enhancers.

Thus, we can make use of T-DNA insertion activation tagging to study the association between genetic function and morphological traits (Hsing et al., 2007). However, there has been no basis for determining whether a target gene is activated by the enhancer prior to experimental analyses. There has even been a study indicating that the enhancer can activate genes that are millions of base pairs away from the enhancer (Li et al., 2012). Not all of the genes that flank the T-DNA insertion site are expected to be activated by the 35S enhancer. In some T-DNA mutants, the 35S enhancer does not activate the closer gene but instead activates a gene that is farther away from the 35S enhancer (Ren et al., 2004). Researchers thus cannot rely on the distance between the enhancer and a particular gene to judge whether that gene would be activated. They must instead determine the activated genes experimentally to explore the related genetic function and morphological traits. Therefore, it is a time-consuming and laborious process to check for the expression of a target gene.

Our team had developed a website platform, EAT-Rice (Liao et al., 2019), for predicting the expression status of rice genes that flank the T-DNA insertion site in activating mutants. In this study, we used a machine learning approach to predict target gene expression in rice T-DNA insertion mutants and improved the efficiency of finding activated target genes. The system of EAT-Rice applied the distance factor from T-DNA insertion site to gene loci to weight feature encoding and used two kinds of algorithms to build a two-layer model of machine learning. Based on EAT-Rice with a modified sequence capturing method, system architecture, and other additional features, we built a more comprehensive system for target gene expression prediction in T-DNA insertion mutants.

The datasets used in this study were experimentally validated. We first characterized genes based on their activation by the 35S enhancer; these genes were divided into activated genes and nonactivated genes. The system we built refers to EAT-Rice. We captured the DNA sequence of the promoter and the central region of each activated gene from the start codon of the target gene to the 35S enhancer and used nine features—CpG islands (CGIs), Motif, Kmer, reverse complementary kmer (RevKmer), DNP, TNP, DACC, TACC, and PseKNC—for encoding. Moreover, we carried out a logistic regression to weight the features of the first-layer model, depending on the probability of gene activation and the distance from the enhancer to the gene start codon. We then used LIBSVM (Chang and Lin, 2011) and LADTree (Boros et al., 2011) algorithms to build a two-layer model of machine learning. In the second layer, we used the minimum redundancy maximum relevance (mRMR) (Peng et al., 2005) method and incremental feature selection to determine the most relevant features. This system is referred to as TIMgo.

The TIMgo performance was 99.3% based on fivefold cross-validation and 85.6% based on independent testing. TIMgo had >80% accuracy for target genes within 20 kb from the 35S enhancer, but genes that were >20 kb away were still predicted with >60% accuracy. We also discovered that the value of the *k* parameter for Kmer, RevKmer, and PseKNC encoding within the PROMOTER sequences was higher than that of MIDDLE sequences. This suggested that for the analysis of longer sequences, a greater number of features was needed to improve the prediction performance. Finally, the G-box cis-element has an important function in gene activation by the 35S enhancer based on the motif analysis, and among the G-box-associated binding proteins, most are bZIP (basic region/leucine zipper) transcription factors.

## 2 Materials and Methods

### 2.1 Sources for T-DNA Mutant Data and Datasets

The experimental data were collected from 11 rice T-DNA mutants from Liang-Jwu Chen’s laboratory at NCHU and 316 mutants from Su-May Yu’s research team at Academia Sinica. These data consisted of the T-DNA insertion point and expression status of flanking genes [as detected by RT-PCR (Ohan and Heikkila, 1993)]. The expression status of each gene was characterized based on the following four categories: activated gene (Ac), no significant effect (NE), non-detectable (ND), and knockout (Ko). The data distribution for the expression status of these genes is shown in Table 1.

**TABLE 1:** Data distribution of flanking analyzed genes in rice T-DNA mutants.

Data source	Number of mutant lines	Gene expression status				Validated genes^a^
		Ac	NE	ND	Ko	
NCHU^b^	11	26	22	17	0	65
Academia Sinica^c^	316	262	143	13	2	420
Total	327	288	165	30	2	485

Ac, activated gene; NE, nonactivated gene; ND, non-detectable gene; Ko, knockout gene.

a Validated genes indicate the target genes that were detected by RT-PCR.

b NCHU, experimental data were collected from Liang-Jwu Chen’s laboratory.

c Academia Sinica, experimental data were collected by Su-May Yu’s research team.

To maintain dataset quality and consistency, we removed the 30 ND genes from the dataset. The collected data included two Ko genes, in which the T-DNA insertion point was located inside the gene, thus disrupting the gene structure and most likely leading to a loss of function. Because Ko genes were not a focus of this study, we removed them from the dataset. We defined NE genes as nonactivated (NAc) genes to differentiate them from the Ac genes. Ultimately, data for 453 genes were collected in this study.

A training set was used to determine the performance of the subsequent system. As the ratio of positive data (Ac genes) to negative data (NAc genes) affects the performance of machine learning (Akbani et al., 2004), this study used EAT-Rice with a 1:1 ratio to carry out the selection of the training dataset. We used data from 300 genes in the training dataset, which was referred to as D300. Data from the remaining 153 genes were used for independent testing to evaluate system accuracy; this dataset was referred to as D153 (Table 2).

**TABLE 2:** Data distribution of the training dataset and independent-testing dataset.

Data sources	Training dataset (D300)		Testing dataset (D153)	
	Ac	NAc	Ac	NAc
NCHU	20	20	6	2
Academia Sinica	130	130	132	13
Total	150	150	138	15

### 2.2 Target Gene Sequence Retrieval

The analyzed genes provided from Liang-Jwu Chen’s laboratory and Su-May Yu’s team were annotated according to the Rice Genome Automated Annotation System (RiceGAAS) (Sakata et al., 2002) and the MSU Rice Genome Annotation Project (TIGR) (Yuan et al., 2003; Ouyang et al., 2007) rice gene annotation database. We hypothesized that we could predict the expression status of a target gene by analyzing the sequence of Ac and NAc genes. Thus, with reference to the EAT-Rice construction process and the enhancer-related hypothesis mechanisms (Singer et al., 2010; Singer et al., 2011), we extracted nucleotide sequences for each gene from two regions: (1) a 1,500-bp region upstream relative to the translation start site (TLS), referred to as the PROMOTER region, and (2) a central region of 300 bp centered between the TLS of the target gene and the 35S enhancer, referred to as the MIDDLE region (Supplementary Figure S1).

### 2.3 Feature Encoding

In this study, we encoded information about nine features of the sequences: five sequence information codes and four biological functional codes. The sequence codes consisted of two local sequence codes, two global sequence codes, and a code to reflect both the local and global sequence information simultaneously. The local sequence characteristics consisted of Kmer and RevKmer values, which were coded by the DNA composition; such characteristics have been successfully applied toward human gene regulatory sequence prediction (Noble et al., 2005; Gupta et al., 2008) and enhancer identification (Lee et al., 2011), among others. The two global sequence codes, dinucleotide-based auto-cross covariance (DACC) and trinucleotide-based auto-cross covariance (TACC), were coded by calculating the sequence autocorrelation as global sequence characteristics; this type of feature has been used to predict sequence-based protein–protein interactions (Guo et al., 2008). Another coding method, PseKNC, has been used to identify promoters in prokaryotes (Lin et al., 2014) and incorporates the information of contiguous local sequence order and the global sequence order into the feature vector. The biological characteristics included the presence of CGIs, regulatory cis-elements (Motif), and conformational and physicochemical properties of dinucleotide and trinucleotide sequences (DNP and TNP, respectively). Each of these features is described in more detail below.

#### 2.3.1 CGIs

DNA methylation on CGIs reduces or silences gene expression based on enhancer–promoter interactions (Antequera et al., 1990; Volpe et al., 2002). For this analysis, we used the EMBOSS Newcpgreport tools of EMBL-EBI to predict CGIs and encoded their corresponding number, length, distance from the TLS, CpG ratio, and OE (observed/expected) value, resulting in the feature CGIs (Supplementary Equations S1–S5).

#### 2.3.2 Regulatory *Cis*-Elements (Motif)

Considering that the rice transcription factor binding sites (TFBSs) that have been confirmed may not be comprehensive enough yet, we therefore incorporated other proven plant TFBSs. Data for 2,087 motifs were collected from PLACE (Higo et al., 1999) and the RegSite database (http://linux1.softberry.com/berry.phtml?topic=regsitelist). The tool Find Individual Motif Occurrences (FIMO) (Grant et al., 2011) in the MEME suite was used to scan for regulatory sequences in the PROMOTER region, and the scanning results were encoded by FIMO (Beer and Tavazoie, 2004; Yuan et al., 2007). These types of feature encoding are referred to as follows.

$$\mathrm{Motif}\_\mathrm{Numbe}r_{\left(i\right)}=\{\begin{array}{c} j,\;\;j\in \;N\\ 0,\;\;\mathrm{otherwise}\;\;\;\; \end{array},\;\;i\in \left\{1,2,\cdots ,2087\right\}$$ (1)

$$\mathrm{Motif}\_\mathrm{Conserv}e_{\left(i\right)}=\frac{M_{i}\;alignment\;score\;in\;promoter}{Motif\_Number\left(i\right)}$$ (2)

$$Motif\_Orientation_{\left(i\right)}=\frac{pos\;in\;Motif\_Number_{\left(i\right)}}{Motif\_Number_{\left(i\right)}}$$ (3)

$$Motif\_Dis_{\left(i\right)}=\frac{\left|geneTLS\;-\;Motif\;location\;site\right|}{Motif\_Number_{\left(i\right)}}$$ (4)

The number of regulatory elements was coded by the number (*j*) of motifs found in the PROMOTER (Equation 1). The conservation score was calculated by FIMO; we used the value from the summed motif conserved scores divided by the number of motifs in the sequence (Equation 2). As motifs can be located on both the DNA coding strand (codons) and the template strand (anticodons), the orientation characteristic was calculated to determine the proportion of motifs on the coding strand. We thus used the number of motifs on the coding strand (i.e., positive motifs, pos) as the numerator, and the denominator is the number of all motifs (Equation 3). The distance characteristic was determined based on the distance (in base pairs) from each motif to the TLS, which was summed for all motif sites within a given sequence, divided by the number of motifs (Equation 4). In these equations, *i* indicates the kinds of motifs, *M*_*i*_ indicates a specific motif, and *geneTLS* refers to the translation start site of a target gene.

#### 2.3.3 Kmer and RevKmer

Kmer refers to the local sequence information and indicates a subsequence containing *k* neighboring nucleic acids in a DNA sequence. Using a coding strand as the template, the Kmer feature will scan for the number of occurrences of the nucleic acid subsequence in the template. For example, when *k* is 2, the subsequence composition of a Kmer will be called a 2-mer, which contains 16 subsequences (based on the four nucleotides G, A, T, and C). In the case of the dinucleotide AA, if this subsequence appeared twice in the DNA template, it would be encoded as 2; if it was not present in the template, it would be encoded as 0. In eukaryotes, the average length of TFBSs is 10 bp (Stewart et al., 2012), which suggests that the number of *k* neighboring nucleic acids in this study could be increased. We encoded the sequence with 3- to 6-mer, 3- to 7-mer, 3- to 8-mer, and 3- to 9-mer, which produced 5,440, 21,824, 87,360, and 349,504 different nucleotide compositions, respectively. The Kmer encoding was carried out based on the number of occurrences in the template sequence (Supplementary Equation S6).

RevKmer is a variant of kmer, in which the kmers are not expected to be strand specific, so reverse complements are collapsed into a single value. In this study, the RevKmer feature was encoded in the same manner as Kmer and produced 2,760, 10,952, 43,848, and 174,920 nucleotide compositions for the 3- to 6-mer, 3- to 7-mer, 3- to 8-mer, and 3- to 9-mer, respectively. RevKmer encoding was carried out according to the number of occurrences in the template sequence (Supplementary Equation S7).

#### 2.3.4 Nucleotide Conformational and Physicochemical Properties (DNP and TNP)

The nucleotide conformation and physicochemical properties of dinucleotides and trinucleotides were also encoded. DiProDB provides information about 125 properties of dinucleotides, and these 125 properties were integrated into 15 characteristics through a statistical principal components analysis (PCA) method (Friedel et al., 2009). The value of each property is based on the dinucleotide as a unit, and each property has 16 values corresponding to all possible dinucleotide combinations. We used the property of the dinucleotide to produce a training model with 240 dimensions; this feature is referred to as the DNP (dinucleotide conformation and physicochemical properties) (Supplementary Equation S8). PseKNC-General (the general form of pseudo *k*-tuple nucleotide composition) is a tool that provides the conformation and physicochemical properties of oligonucleotides (Chen et al., 2015). In this study, 12 trinucleotide properties were used for coding. There were 64 combinations of trinucleotides, which generated a training model with 768 dimensions based on the 12 trinucleotide properties; this feature is referred to as the TNP (trinucleotide conformation and physicochemical properties) (Supplementary Equation S9).

#### 2.3.5 Autocorrelation (DACC and TACC)

Pse-in-One provides a pseudo-component mode reflecting the correlation between two dinucleotides or trinucleotides within a DNA sequence via their physicochemical properties (Liu et al., 2015). In this study, we used dinucleotide-based auto-cross covariance (DACC) and trinucleotide-based auto-cross covariance (TACC) as provided by Pse-in-One for encoding (Supplementary Equations S10–S12).

In this study, DACC was based on the 15 properties from DiProDB, and the lag value was 4, generating a training model with 900 dimensions. TACC used the 12 Pse-in-One built-in properties, and the lag value was 4; it generated a training model with 576 dimensions.

#### 2.3.6 Pseudo k-Tuple Nucleotide Composition

Pseudo k-tuple nucleotide composition (PseKNC) is one of the encoding modes supplied by Pse-in-One. It incorporates both the contiguous local sequence order information (like Kmer and RevKmer) and the global sequence order information (like DACC and TACC) into the feature vector of the DNA sequence.

$$D=R_{1}R_{2}R_{3}R_{4}R_{5}R_{6}\cdots R_{L}$$ (5)

$$\mathrm{PseKN}C_{\left(u\right)}=\{\begin{array}{c} \frac{f_{u}}{\sum_{i=1}^{4^{k}}f_{i}+w\sum_{j=1}^{\lambda }\theta_{j}},u\in \left\{1,2,\cdots 4^{k}\right\}\\ \frac{w\theta_{u-4^{k}}}{\sum_{i=1}^{4^{k}}f_{i}+w\sum_{j=1}^{\lambda }\theta_{j}},u\in \left\{4^{k+1},\left(4^{k+1}+1\right),\cdots ,\left(4^{k+1}+\lambda \right)\right\} \end{array}$$ (6)

$$\theta_{j}=\frac{1}{L-j-1}\overset{L-j-1}{\underset{i=1}{\sum }}\left\{\frac{1}{\mu }\overset{u}{\underset{v=1}{\sum }}{\left[P_{v}\left(R_{i}R_{i+1}\right)-P_{v}\left(R_{i+j}R_{i+j+1}\right)\right]}^{2}\right\},j\in \left\{1,2,\cdots ,\lambda \right\},\lambda <L$$ (7)

For a DNA sequence D with *L* nucleic acid residues, R_1_ represents the nucleic acid residue at the sequence position 1, R_2_ the nucleic acid residue at position 2, and so on (Equation 5). PseKNC will calculate the occurrence frequency (*f*) of dinucleotides in the DNA sequence and the correlation between two oligonucleotides that are 1 to *λ* nucleotides apart from each other. In Equation 6, *f*_*u*_ is the occurrence frequency of dinucleotides in the DNA sequence, which is normalized to $\sum_{i=1}^{4^{k}}f_{i}=1$ ; *w* is the weight factor; *θ*_*j*_ represents the correlation factor that reflects the sequence-order correlation between all two dinucleotides that are *j* nucleotides away from each other along a DNA sequence; *µ* is the number of physicochemical indices; *P*_*v*_(*R*_*i*_ *R*_*i*+1_) represents the numerical value of the dinucleotide located at the *i*th position (*R*_*i*_ *R*_*i*+1_) of the *v*th (*v* = 1, 2, …, μ) physicochemical property (Equation 7). The feature number of PseKNC will be *λ* multiplied by 4 to the power *k*. In this study, the PseKNC feature was determined with a *λ* value of 4, *w* is 0.2, and *k* is from 2 to 6.

### 2.4 Significant Sequence Fragment Analysis

Because there are numerous features in this first-layer model, the complexity of the model is relatively high. To reduce the interference of excessive noise, we used independent two-sample *t*-tests (implemented in R) to select features from the high-dimension models. We used the occurrence of specific oligonucleotides in the Ac and NAc groups to generate the *t*-test (Supplementary Figure S2) and retained the oligonucleotides with *p* < 0.05 to encode these significant fragments.

### 2.5 Model Evaluation and Cross-Validation

We used a five-fold cross-validation method and independent-testing data to evaluate the predictive performance of the model. Our evaluation methods included accuracy (Acc), sensitivity (Sn), specificity (Sp), and Matthews correlation coefficient (MCC). Acc is used to estimate the prediction accuracy of the global prediction capability, with values closer to 100% indicating better overall predictive performance of a model (Equation 8). Sn and Sp evaluate the accuracy of the prediction of positive and negative data, respectively (Equations 9 and 10). When the number of positive and negative data differs, Acc is not a good evaluation indicator. MCC is, however, suitable for assessing a dataset in which there is an imbalance between positive data and negative data (Equation 11). When the MCC score is closer to 1, the prediction capability is better; a score closer to −1 indicates a worse prediction capability.

$$\mathrm{Acc}=\frac{TP+TN}{TP+FP+TN+FN}$$ (8)

$$\mathrm{Sn}=\frac{TP}{TP+FN}$$ (9)

$$\mathrm{Sp}=\frac{TN}{TN+FP}$$ (10)

$$\mathrm{MCC}=\frac{\left(TP\times TN\right)-\left(FN\times FP\right)}{\sqrt{\left(TP+FN\right)\left(TN+FP\right)\left(TP+FP\right)\left(TN+FN\right)}}$$ (11)

### 2.6 Framework of TIMgo

**FIGURE 1:**
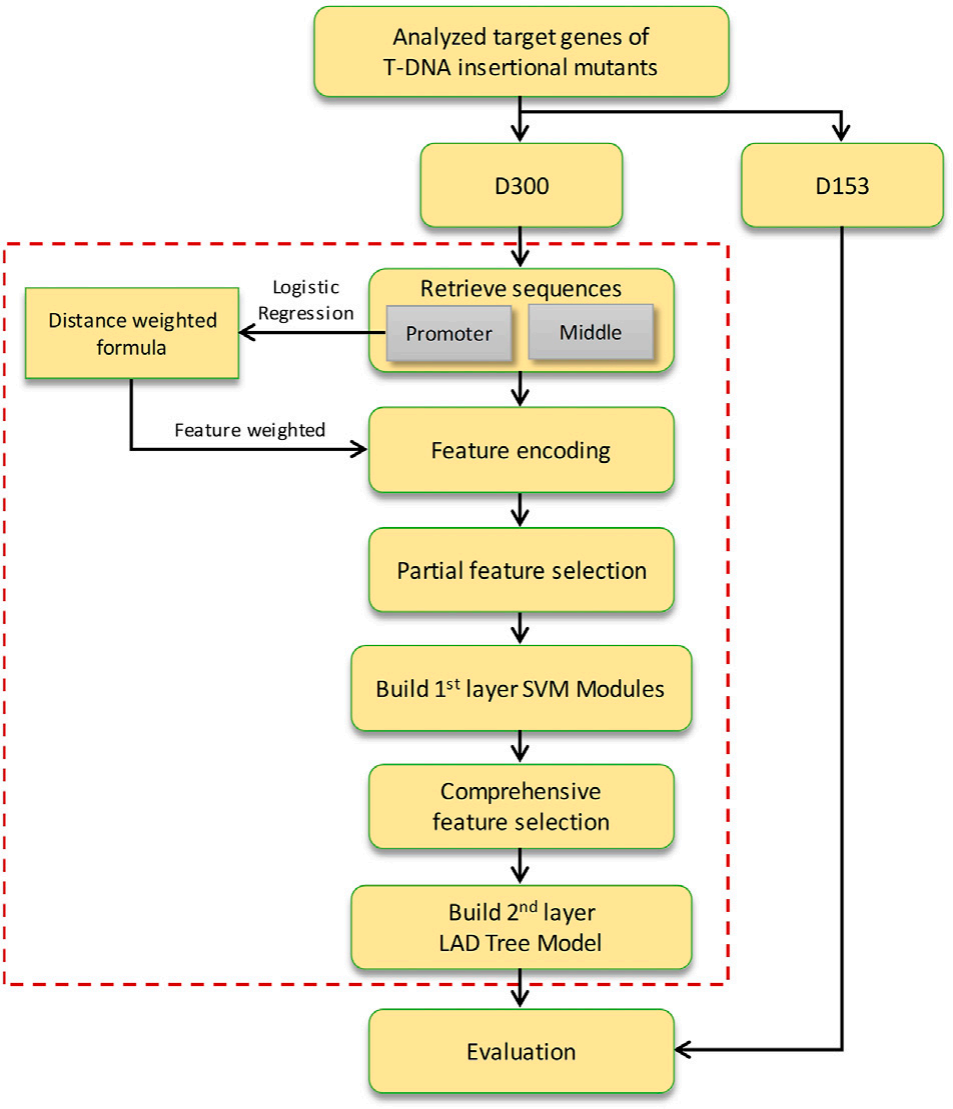
Flow chart of the TIMgo predictive system.

TIMgo is a two-layer machine learning model constructed for predicting the effect of a 35S enhancer on the expression of the target gene (Figure 1). The D453 was divided into a training dataset (D300) and independent testing data (D153). The DNA sequences of PROMOTER and MIDDLE were retrieved for analysis between NAc and Ac genes. In the first-layer module, the support vector machine (SVM) models were constructed within nine feature-encoding methods. And the significant sequences were analyzed by Student’s *t*-test, and a model of logistic regression was used to assist in training, which is based on the relationship between distance from the 35S enhancer to the target gene and states of gene expression. The features encoded from the PROMOTER region were weighted by a logical regression model for probability of gene activation. Then, we adopted feature selection by the LIBSVM built-in tool in the partial SVM models. The prediction results of the first-layer module were integrated into the second-layer model, and mRMR (Peng et al., 2005) was used for feature selection and building the LAD tree model. Finally, we evaluated the prediction efficacy of TIMgo with the D153 independent-testing dataset.

## 3 Results

### 3.1 Correlation Between Gene Activation and Distance From the 35S Enhancer to the TLS

**FIGURE 2:**
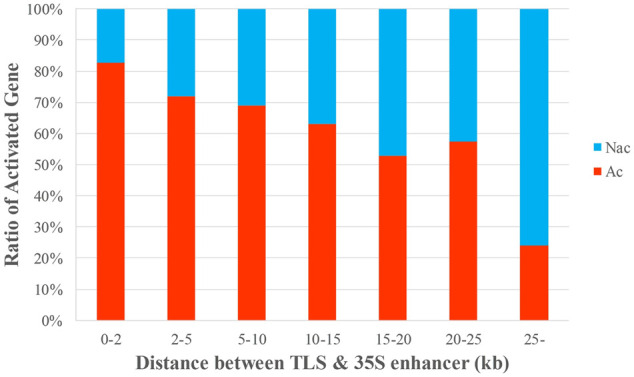
Correlation between distance and gene activation. The data were sorted by the distance between the 35S enhancer and the TLS, and the ratio of Ac to NAc genes in each group was calculated. The *x*-axis is the distance from the 35S enhancer to the TLS of a target gene; the *y*-axis is the proportion of Ac and NAc genes in each group.

The distance between the enhancer and a target gene cannot be directly used to determine whether the target gene will be activated, although it does have some relevance for determining gene activation (Vandergeest and Hall, 1997; Jagannath et al., 2001). A target gene is more likely to be activated if it is closer to the enhancer (Marenduzzo et al., 2007). We characterized each of the 453 genes in the entire dataset (D453) based on the distance from the CaMV 35S enhancer on the inserted T-DNA to TLS and calculated the ratio of Ac genes and NAc genes. We found a negative correlation between this distance and gene activation. Genes closer to the 35S enhancer had a greater probability of activation (*p* < 0.001) (Supplementary Figure S3). The results are the same as those indicated in a previous study (Liao et al., 2019) (Figure 2, Supplementary Table S1).

Among the D453 dataset, there were 94 sets of duplicated data which consist of multiple genes, and the PROMOTER sequences corresponding to these genes were identical. Each of the experimental data in this study represented the effect of a single insertion event on its target gene. In the experimental data collected in this study, when the same gene was detected for multiple T-DNA insertion events, the PROMOTER sequences from those genes were identical. For different T-DNA insert events, the 35S enhancer may result in different states of expression for the same target gene, which will lead to contradictory results while building the machine learning model. To distinguish between these PROMOTER sequences, we used logistic regression to build a regression model of the distance coefficient and the target gene activation probability (Supplementary Equation S13). In this study, the values calculated by logistic regression were used to weight the promoter sequence feature, so that the same sequence could be distinguished when quantified based on numerical values.

### 3.2 Comparison of Kmer and RevKmer Combined With Motif

In the Kmer and RevKmer feature models, a *t*-test was used to calculate the number of occurrences of specific sequence fragments in Ac and NAc genes, respectively, from sequence lengths (*k*) of three to nine nucleotides. The specific sequence fragments with *p* < 0.05 were then used for encoding. These fragments were combined as 3–6, 3–7, 3–8, and, 3–9 combinations for Kmer and RevKmer. The Motif feature was used to carry out a similar analysis. The Kmer and RevKmer features associated with the PROMOTER region were combined with the Motif feature (Supplementary Table S2). The features from Kmer, RevKmer, Kmer + Motif, and RevKmer + Motif were used to build SVM models, and the best model was selected for the second-layer model integration (Supplementary Table S3).

Before combining Motif with Kmer or RevKmer, the Acc scores of the SVM models of Kmer and RevKmer were 55%–85%, whereas the Acc scores of the Motif models were 52%–75%. After combining Motif with Kmer or RevKmer, the Acc scores were 78%–86%, and the Acc consistently increased with the *k* value for Kmer and RevKmer (Table 3).

**TABLE 3:** Data distribution of the training dataset and independent-testing dataset.

Feature	*k* ^a^	Without motif					With motif				
		Sp (%)	Sn (%)	Acc (%)	MCC (%)	AUC (%)	Sp (%)	Sn (%)	Acc (%)	MCC (%)	AUC (%)
Kmer	6	72.7	66.0	69.3	38.8	79.0	79.3	77.3	78.3	56.7	88.1
	7	86.7	73.3	80.0	60.5	89.1	83.3	78.7	81.0	62.1	89.7
	8	75.3	35.3	55.3	11.6	65.3	83.3	84.7	84.0	68.0	93.6
	9	84.7	85.3	85.0	70.0	93.2	86.7	85.3	86.0	72.0	93.7
RevKmer	6	71.3	60.7	66.0	32.2	72.7	78.0	77.3	77.7	55.3	85.7
	7	84.7	76.0	80.3	60.9	87.9	79.3	77.3	78.3	56.7	88.1
	8	77.3	32.7	55.0	11.2	64.9	84.0	80.0	82.0	64.1	91.5
	9	74.7	88.0	81.3	63.2	90.6	84.0	84.7	84.3	68.7	92.9

a *k* refers to the maximum *k* value used in Kmer and RevKmer, with a range of 3-*k* nucleotides in length for each analysis.

### 3.3 First-Layer Model Evaluation

In the first-layer models, nine feature coding methods and two types of sequences were used to construct 16 feature models (Supplementary Table S4). The prediction ability of each feature model was evaluated with fivefold cross-validation and independent testing with the D153 data (Table 4). For the Pse-in-One feature encoding, one gene sequence from the training dataset (D300) did not conform to the encoding requirements. Therefore, in the DACC, TACC, and PseKNC models, this information was removed from the training data, and the training dataset consisting of the remaining 299 genes was referred to as D299. The PseKNC models used *k* values of 2–7, and eight models each were established for the PROMOTER and MIDDLE sequences. A PseKNC model with *k* = 6 that was selected among the PROMOTER models had an Acc of 75.3% with fivefold cross-validation. The PseKNC model with *k* = 2 that was selected among the MIDDLE models had an Acc of 59.5% (Supplementary Table S5).

**TABLE 4:** Performance of the first-layer features with the SVM models.

Feature encoding	Sequence	Cross-validation					Independent testing				
		Sp (%)	Sn (%)	Acc (%)	MCC (%)	AUC (%)	Sp (%)	Sn (%)	Acc (%)	MCC (%)	AUC (%)
CGIs	PROMOTER	71.3	48.7	60.0	20.5	58.5	53.3	40.6	41.8	−3.7	48.2
	MIDDLE	77.3	18.0	47.7	−5.8	47.2	100.0	2.2	11.8	4.7	65.0
DNP	PROMOTER	56.0	64.7	60.3	20.7	64.3	26.7	71.7	67.3	−1.1	45.1
	MIDDLE	59.3	62.0	60.7	21.3	60.0	60.0	53.6	54.3	8.1	48.7
TNP	PROMOTER	56.0	61.3	58.7	17.4	62.2	53.3	68.1	66.7	13.5	57.4
	MIDDLE	64.7	30.0	47.3	−5.7	47.4	26.7	65.9	62.1	−4.7	45.0
Kmer + Motif	PROMOTER	86.7	85.3	86.0	72.0	93.7	73.3	85.5	84.3	43.5	79.1
RevKmer + Motif	PROMOTER	84.0	84.7	84.3	68.7	92.9	73.3	81.2	80.4	37.8	83.6
Kmer	MIDDLE	92.0	84.7	88.3	76.9	94.2	66.7	86.2	84.3	40.1	86.4
RevKmer	MIDDLE	85.3	72.7	79.0	58.5	88.2	53.3	68.8	67.3	14.0	66.5
DACC	PROMOTER	67.1	72.7	69.9	39.8	78.6	46.7	59.4	58.2	3.7	54.6
	MIDDLE	76.5	58.0	67.2	35.1	74.1	53.3	49.3	49.7	1.6	47.5
TACC	PROMOTER	60.4	58.0	59.2	18.4	60.3	13.3	63.0	58.2	−14.8	41.6
	MIDDLE	59.7	56.7	58.2	16.4	57.8	46.7	45.7	45.8	−4.6	45.1
PseKNC	PROMOTER	89.9	60.7	75.3	52.9	84.5	73.3	54.3	56.2	16.5	59.1
	MIDDLE	56.4	52.7	59.5	19.1	61.7	66.7	58.0	58.8	14.7	54.5

In the evaluation results of the first-layer feature models (Table 4), the Kmer, RevKmer, Kmer + Motif, and RevKmer + Motif had the best predictive performance based on the Kmer feature provided. Their Acc values were 79.0%–88.3% with fivefold cross-validation. With independent testing, their Acc values were 80.4%–84.3%, with the exception of RevKmer, which had 67.3%. The PseKNC model built using the PROMOTER sequence was slightly inferior to the model built using Kmer-related features. The Acc and MCC values for PseKNC were 75.3% and 52.9% with cross-validation, respectively, and 56.2% for Acc and 16.5% for MCC with independent testing. The DACC, TACC, DNP, CGIs, and TNP constructed by the PROMOTER sequence and the PseKNC constructed by the MIDDLE sequence had lower predictive performance, with Acc values of 58.2%–69.9% and MCC values of 16.4%–39.8%. Among these 16 models, CGIs and TNP constructed using the MIDDLE sequence were the least accurate in cross-validation, with an Acc of ∼47%. Their Acc values for independent testing were 11.8% and 62.1%, respectively. In terms of overall predictive performance, the PROMOTER sequence is thus more important than the MIDDLE sequence, and Kmer, RevKmer, Kmer + Motif, and RevKmer + Motif features have the highest correlation with the activation of genes.

### 3.4 Comprehensive Feature Selection in the Second-Layer Model

**FIGURE 3:**
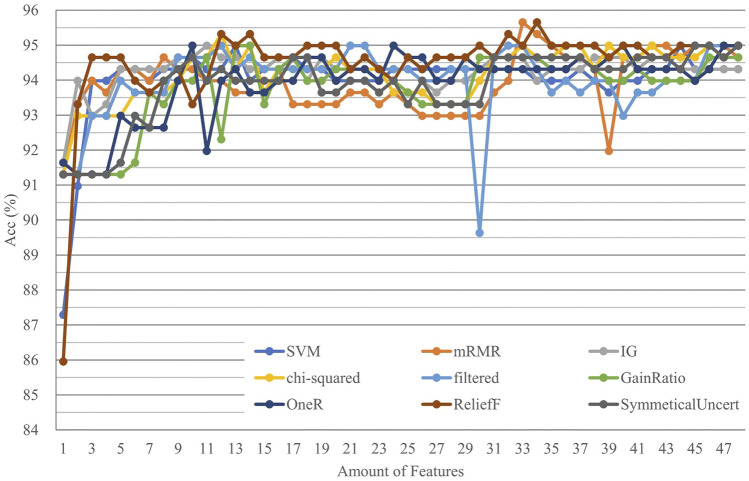
Accuracy trend in the second-layer feature selection.

The second-layer model integrated the prediction results from the 16 feature models in the first layer and obtained the ultimate prediction result by machine learning. The features used in the second-layer model of this study included predictive results and positive and negative predictive confidence scores, generating 48 features. We used incremental feature selection and an SVM model with cross-validation to carry out comprehensive feature selection among these 48 features to pick out the best feature combinations with nine feature selection methods. The top 33 features of the mRMR (Peng et al., 2005) were selected as the best feature combination with the highest Acc and the fewest features (Figure 3, Supplementary Table S6). Among the 33 selected features, we knew that the encoding contributed for classification is Kmer related, DACC was better than PseKNC and TACC, and CGIs, TNP, and DNP are worse.

### 3.5 Second-Layer Model Evaluation

We assessed the best-suited machine learning algorithm for the second-layer model through the WEKA (Holmes et al., 1994) analysis platform. In this study, we used the 65 algorithms provided by WEKA to establish the model separately and evaluated the effectiveness of these models with cross-validation (Supplementary Table S7). In this experiment, the LADTree algorithm was used to construct the second-layer integration model according to the above conditions. The Acc was 99.3%, MCC was 98.7%, and Sn and Sp were 99.3%. In independent testing, the model Acc reached 85.6%, MCC was 35.3%, Sn was 89.1%, and Sp was 53.3%. Among the testing data, there were only 15 negative data, such that each predictive result with these data would lead to a substantial impact on the overall predictive effectiveness assessment. Among these models built with multiple algorithms, Sp values ranged from 46.7% to 73.3%, which corresponded to a difference of only six correctly predicted negative data.

### 3.6 Correlation Between Predictive Accuracy and Distance From the 35S Enhancer to TLS

**FIGURE 4:**
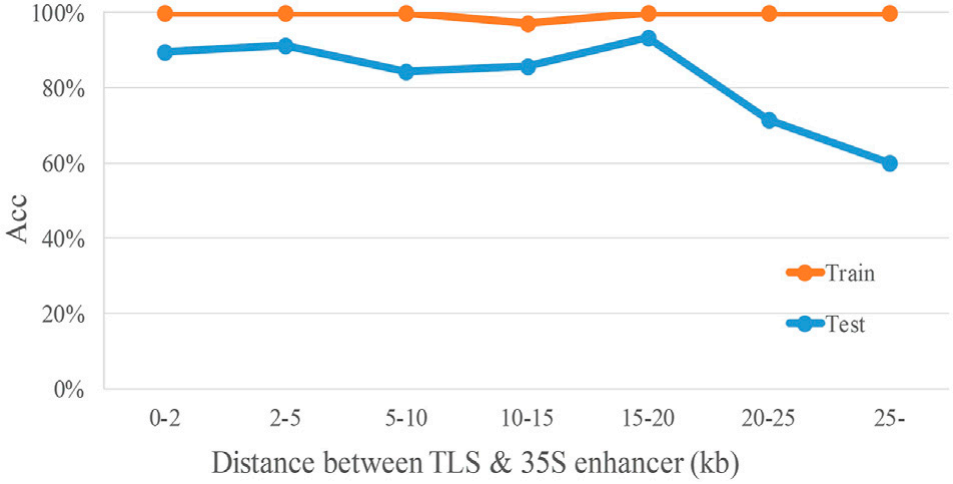
Accuracy trend of TIMgo for cross-validation and independent testing of data within different distances. Train represents the Acc from fivefold cross-validation with D299. Test represents the Acc from independent testing with D153. The *x*-axis indicates each distance interval, and the *y*-axis indicates the predictive accuracy.

To analyze the relationship between distance and TIMgo prediction accuracy, the training dataset and independent-testing dataset were grouped according to the distance between the TLS and 35S enhancer (Figure 4). In cross-validation, Acc was 99.3%, and predictions for only two genes were incorrect (Table 5); these two genes were 10–15 kb away from the 35S enhancer. In independent testing, the prediction accuracy for genes within 20 kb from the 35S enhancer was >84%. For genes located >20 kb from the 35S enhancer, the prediction accuracy decreased with increasing distance but still was >60% (Table 6).

**TABLE 5:** Performance of the LADTree model in the second-layer.

	TP	FP	TN	FN	Sn (%)	Sp (%)	Acc (%)	MCC (%)
Cross-validation	149	1	148	1	99.3	99.3	99.3	98.7
Independent testing	123	7	8	15	89.1	53.3	85.6	35.3

**TABLE 6:** Predictive accuracy of TIMgo for different distance groups.

Distance from the 35S enhancer (kb)							
Dataset	0–2	2–5	5–10	10–15	15–20	20–25	>25
Training set	100.0%	100.0%	100.0%	97.0%	100.0%	100.0%	100.0%
Testing set	89.0%	91.0%	84.0%	86.0%	93.0%	71.0%	60.0%

## 4 Discussion

### 4.1 Comparison of the Framework Between TIMgo and EAT-Rice

In a previous study, the PROMOTER region for most genes was defined as the upstream region from the transcription start site (TSS) (Chang et al., 2008). For the EAT-Rice analysis, however, as the collected gene data had information about only the TLS, the PROMOTER region, including the upstream sequence of the TSS, was based on a 1,000-bp region upstream of the TLS. The upstream sequence of the TSS contains the 5′ untranslated region of the mRNA, and sequences downstream of the TSS may also be involved with transcription factor regulation of gene expression (Heyndrickx et al., 2014). Given an average length of 500 bp for 5′ untranslated regions in rice and the 1,000 bp upstream of the TSS as the condition, we used the 1,500-bp sequence upstream of the TLS as the PROMOTER region in this study.

For our prediction models, we retained the EAT-Rice CGIs and DNP (dinucleotide conformation and physicochemical properties encoding) and increased the TNP coding with the DNP coding concept. We also used the Pse-in-One tool to generate codes for DACC, TACC, and PseKNC. Given the strand specificity of Kmer, we added RevKmer coding, and the Motif coding of the PROMOTER region was combined with Kmer and with RevKmer. The ranges of overall predictive accuracy for Kmer + Motif and RevKmer + Motif models were small, which indicated that Motif was complementary with Kmer and RevKmer, and the combination of these two features could improve the classification ability. Predictive accuracy increased with the length of *k* for both Kmer and RevKmer, because that Motif feature consisted of experimentally validated regulatory sequences, but the number of proven regulatory sequences in plants is limited, whereas Kmer and RevKmer considered all the sequence combinations that provided higher data integrity than Motif, so using longer Kmer and RevKmer should lead to better prediction performance. Although Kmer and RevKmer had higher data integrity than Motif, the complexity of the Kmer and RevKmer data increased exponentially with the increase in sequence length, resulting in processing time that was too lengthy. Therefore, we used Kmer (and RevKmer) with limited *k* length and retained Motif with longer sequences, to preserve important regulatory sequence data and reduce the computational complexity significantly.

### 4.2 Specific Regulatory Sequences Within Genes Activated by the 35S Enhancer

To find out whether a specific regulatory sequence was related to gene activation in the T-DNA insertion mutants, we analyzed the 2,087 motifs with a *t*-test. We found that there were 181 regulatory sequences that had significant difference in their occurrence frequency between Ac and NAc genes. Among these 181 regulatory sequences, 20 were G-box and G-box-related sequences. The G-box contains a core region, CACGTG, and flanking sequences that are composed of other nucleotides. The G-box-binding protein has different binding preferences and affinities according to the different flanking sequences in the G-box. bZIP (basic region/leucine zipper) transcription factors account for the majority of G-box-binding proteins. Transcription regulation in plants is often affected by G-box sequences, such as stress hormones (e.g., abscisic acid), seed germination, protein storage, and light response (Marcotte et al., 1989; Donald and Cashmore, 1990; Mason et al., 1993). Thus, the G-box may have important biological significance in the regulation of gene expression by the 35S enhancer and may affect whether the 35S enhancer will activate a target gene in rice.

### 4.3 Correlation Between Length of Sequence and Nucleotide Length Parameter

In the feature coding of TIMgo, the coding of Kmer, RevKmer, and PseKNC can be adjusted based on the nucleotide length parameter (*k*). We needed to find a suitable nucleotide length parameter for encoding. For these three kinds of coding, the *k* value selected for the PROMOTER region was greater than that for the MIDDLE region. A higher value for *k* results in a higher number of features being generated, which requires more features to be improved to increase the predictive accuracy of the PROMOTER region, relative to the MIDDLE region. Thus, an excessive number of features would reduce the predictive performance of the model. From the optimal *k* value for the MIDDLE sequence, we could see that a higher number of features did not necessarily make the classification better. By comparing the optimal *k* value selected for the PROMOTER and MIDDLE regions, we note that a longer sequence does seem to require more features to make the classification better. Moreover, among the local, global, and local + global sequence characteristics used to build the TIMgo, the local sequences had a greater contribution with respect to identifying activation of the target genes (Table 4).

### 4.4 Performance Comparison of TIMgo and EAT-Rice

To confirm that the model constructed by the framework of TIMgo is superior to that of EAT-Rice, the training dataset and testing dataset used to develop EAT-Rice were used to build models in the TIMgo framework and to evaluate TIMgo by comparing their predictive performance. The training dataset used with EAT-Rice had data for 280 validated genes, and these 280 data points were separated into two subsets (subset1 and subset2) with 180 validated genes (Liao, et al., 2019). The independent-testing dataset used with EAT-Rice had 48 validated genes. Two training datasets (subset1 and subset2) were used to build training models within the framework of TIMgo, and the predictive efficacy of EAT-Rice and TIMgo was evaluated with an independent-testing dataset consisting of an additional 48 validated genes (Table 7). With the use of subset1 as the training dataset and of the EAT-Rice system to establish the model, the Acc in the independent testing was 72.9%, the Acc for TIMgo was 79.2%, and the Sp value of TIMgo was 12.8% higher than that of EAT-Rice. With subset2 as the training dataset, the Acc with independent testing was 77.1% for EAT-Rice and 77.6% for TIMgo. In the case of using the same training dataset and testing dataset, the accuracy of the TIMgo framework is better than that of EAT-Rice.

**TABLE 7:** Comparison of TIMgo and EAT-Rice with independent-testing evaluation.

System	Subset1				Subset2			
	Sp (%)	Sn (%)	Acc (%)	AUC (%)	Sp (%)	Sn (%)	Acc (%)	AUC (%)
EAT-Rice	59.1	84.6	72.9	79.4	59.1	92.3	77.1	83.2
TIMgo	72.7	84.6	79.2	87.4	78.3	76.7	77.6	84.4

## 5 Conclusion

In this study, we analyzed the DNA sequence and constructed a two-layer model system using the machine learning method to predict whether the 35S enhancer would affect the expression of a target gene in T-DNA insertion mutants. The first layer of the system was built with the PROMOTER and MIDDLE sequences and was encoded using nine features. We analyzed significant sequence fragments in Motif, Kmer, and RevKmer and weighted the PROMOTER based on a logistic regression analysis of the distance between the 35S enhancer and the TLS of each gene. Some of the first-layer SVM models were built with LIBSVM feature selection. The second-layer model used the mRMR feature selection tool to select the predicted values from the 16 models in the first layer, and these were integrated with the LADTree algorithm as the second-layer model. The predictive performance of TIMgo had Acc of 99.3% and 85.6% with cross-validation and with independent testing, respectively. TIMgo can more accurately predict the activation of genes located within 20 kb of the 35S enhancer. We analyzed the 2,087 motifs and found that there was a significant difference in the frequency of G-box sequences between Ac and NAc genes, suggesting that the G-box may play an important role in the activation mechanism of 35S enhancer genes. Our model has improved the predictive ability of determining target gene activation based on the CaMV 35S enhancer in rice T-DNA insertion mutants.

## Data Availability

The original contributions presented in the study are included in the article/Supplementary Material, further inquiries can be directed to the corresponding author.
