# Supplementary material for: Using Machine Learning Approaches to Predict Target Gene Expression in Rice T-DNA Insertional Mutants
Source: Front Genet. 2021 Dec 17;12:798107. doi: 10.3389/fgene.2021.798107 (PMC8718795; doi:10.3389/fgene.2021.798107)
Supplement: Supplementary file 1 [file Presentation1.PDF]

Supplementary Material

1 Supplementary Figures and Tables

1.1 Supplementary Figures

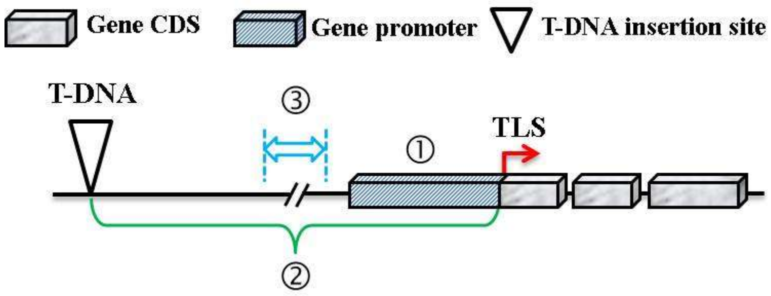

Figure S1. A schematic view of the target gene sequence retrieval. Fragment 1 is the 1500-bp upstream sequence relative to the TLS, which is referred to as the Promoter region. Fragment 2 consists of a 300-bp region centered between the 35S enhancer region and the TLS, which is referred to as the Middle region.

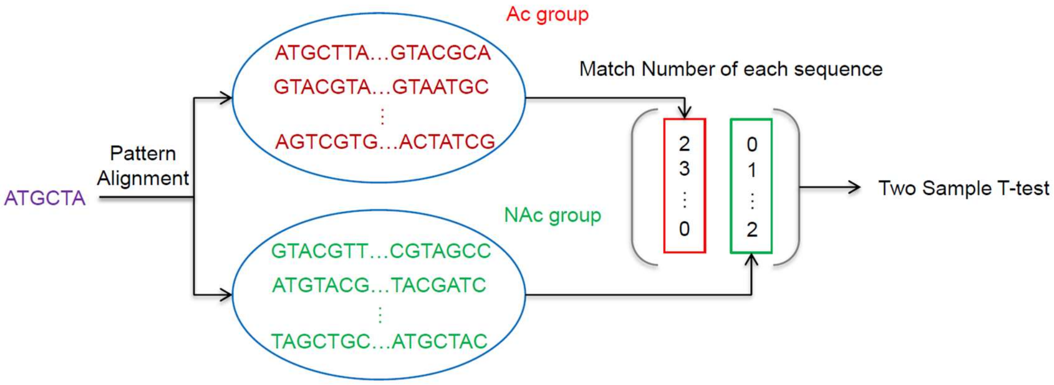

Figure S2. Analysis of significant sequence fragments. The purple sequence represents a specific fragment from Motif, Kmer **or** RevKmer, which is compared to each sequence between the Ac and NAc genes, respectively. The number of specific fragments that were matched in each sequence was divided into two groups according to the expression state of each gene. The results from the t-test indicate whether this specific fragment has a significant difference with respect to its frequency of occurrence among Ac and NAc genes.

```
Call:
glm(formula = Gene.exp. ~ Distance..bp., family = binomial(link = "logit"),
    data = DTrain)

Deviance Residuals:
    Min       1Q   Median       3Q      Max
-1.7774  -1.2565   0.7548   0.9200   1.4239

Coefficients:
                Estimate Std. Error z value Pr(>|z|)
(Intercept)    1.349e+00  1.838e-01   7.339 2.15e-13
Distance..bp. -6.592e-05  1.266e-05  -5.207 1.92e-07

(Intercept)   ***
Distance..bp. ***
---
Signif. codes:
0 '***' 0.001 '**' 0.01 '*' 0.05 '.' 0.1 ' ' 1

(Dispersion parameter for binomial family taken to be 1)

    Null deviance: 594.17  on 452  degrees of freedom
Residual deviance: 561.58  on 451  degrees of freedom
AIC: 565.58

Number of Fisher Scoring iterations: 4
```

Figure S3. Logistic regression analysis report in R language. Distance..bp. is the distance between the CaMV 35S enhancer and the TLS; Pr(> |z|) represents the P-value if the distance is not associated with gene activation.

1.2 Supplementary Tables

Table S1. The number of different interval region is divided into two expression states.

Status \ (kb)	0~2	2~5	5~10	10~15	15~20	20~25	25~	Total
**Ac**	38	49	78	56	38	23	6	288
**NAc**	8	19	35	33	34	17	19	165
**Total**	46	68	113	99	72	40	25	453

Table S2. The number of significant sequences from Kmer, RevKmer and Motif.

**Feature**		**Promoter region**					**Middle region**			
	k[a]	**3–6**	**3–7**	**3–8**	**3–9**		**3–6**	**3–7**	**3–8**	**3–9**
**Kmer**		466	1991	8307	27135		412	1882	6555	11734
**RevKmer**		231	966	4023	16108		208	859	3765	9088
	**Length (nt)**[b]	**6**	**7**	**8**	**9**	**10**				

**Motif**		181	170	156	142	120

[a]*k* Indicates the number of nucleic acids for Kmer and RevKmer
[b]Length means that the length of Motif fragments was longer than or equal to the specified number of nucleic acids

Table S3. Performance of Kmer, RevKmer and Motif in five-fold cross-validation.

Feature	Length (nt)[a]	Promoter region					Middle region				
		Sp	Sn	Acc	MCC	AUC	Sp	Sn	Acc	MCC	AUC
**Kmer**											
	**3–6**	0.727	0.660	0.693	0.388	0.790	0.900	0.667	0.783	0.583	0.874
	**3–7**	0.867	0.733	0.800	0.605	0.891	0.947	0.773	0.860	0.731	0.941
	**3–8**	0.753	0.353	0.553	0.116	0.653	0.920	0.847	0.883	0.769	0.942[b]
	**3–9**	0.847	0.853	0.850	0.700	0.932	0.940	0.773	0.857	0.723	0.949
**RevKmer**											
	**3–6**	0.713	0.607	0.66	0.322	0.727	0.780	0.593	0.687	0.380	0.732
	**3–7**	0.847	0.760	0.803	0.609	0.879	0.873	0.693	0.783	0.576	0.867
	**3–8**	0.773	0.327	0.550	0.112	0.649	0.853	0.727	0.790	0.585	0.882
	**3–9**	0.747	0.880	0.813	0.632	0.906	0.947	0.740	0.843	0.702	0.932
**Motif**											
	**6+**	0.693	0.353	0.523	0.050	0.604					
	**7+**	0.767	0.720	0.743	0.487	0.830					
	**8+**	0.727	0.733	0.730	0.460	0.829					
	**9+**	0.753	0.747	0.750	0.500	0.825					
	**10+**	0.747	0.693	0.720	0.441	0.800					
**Kmer + Motif**											
	**6 + 7**	0.793	0.773	0.783	0.567	0.881					
	**7 + 8**	0.833	0.787	0.810	0.621	0.897					
	**8 + 9**	0.833	0.847	0.840	0.680	0.936					
	**9 + 10**	0.867	0.853	0.860	0.720	0.937					
**RevKmer + Motif**											
	**6 + 7**	0.780	0.773	0.777	0.553	0.857					
	**7 + 8**	0.793	0.773	0.783	0.567	0.881					
	**8 + 9**	0.840	0.800	0.820	0.641	0.915					
	**9 + 10**	0.840	0.847	0.843	0.687	0.929					

[a]Length indicates the fragment length in nucleotides for each feature. Given a number of nucleotides *k*, the length in Motif is shown as *k*+, as motifs that are ≥*k* nucleotides to are encoded. The lengths in Kmer and RevKmer are shown as *k*–*k*', which indicates the subsequence range of neighboring nucleic acids analyzed for Kmer and RevKmer. In Kmer + Motif and RevKmer + Motif, length is represented as *k* + ', and *k* means that Kmer and RevKmer used the subsequences of length 3–*k* combined with a motif length ≥*k*'.

[b]Gray shading indicates the selected combination for the second-layer training model.

Table S4. Feature encoding of two pattern sequences.

Feature encoding	Sequences	
	PROMOTER	MIDDLE
**CGIs**	●	●
**Kmer**	○	●
**Kmer + Motif**	●	○
**RevKmer**	○	●
**RevKmer + Motif**	●	○
**DNP**	●	●
**TNP**	●	●
**DACC**	●	●
**TACC**	●	●
**PseKNC**	●	●

● Indicates that the feature was present in the sequence.
○ Indicates that the feature was not present in the sequence.

Table S5. Performance of PseKNC in five-fold cross-validation.

k	PROMOTER					MIDDLE				
	Sp	Sn	Acc	MCC	AUC	Sp	Sn	Acc	MCC	AUC
**2**	0.651	0.527	0.589	0.179	0.615	0.564	0.527	0.595	0.191	0.617
**3**	0.624	0.653	0.639	0.278	0.674	0.456	0.580	0.518	0.037	0.493
**4**	0.638	0.713	0.676	0.352	0.733	0.503	0.533	0.518	0.037	0.531
**5**	0.785	0.640	0.712	0.430	0.779	0.463	0.427	0.445	–0.110	0.431
**6**	0.899	0.607	0.753	0.529	0.845	0.477	0.473	0.475	–0.050	0.471
**7**	0.705	0.733	0.719	0.438	0.797	0.376	0.527	0.452	–0.099	0.466

Table S6. Feature composition optimization on second layer model.

Feature selection tool	Amount of features	Acc	Sn	Sp	AUC	MCC
**SVM**	13	0.950	0.940	0.960	0.982	0.900
**IG**	11	0.950	0.940	0.960	0.984	0.900
**mRMR**	33	0.957	0.960	0.953	0.986	0.913
**ChiSquared**	12	0.950	0.940	0.960	0.984	0.900
**Filter**	12	0.950	0.940	0.960	0.982	0.900
**GainRatio**	13	0.950	0.933	0.966	0.985	0.900
**OneR**	10	0.950	0.933	0.966	0.984	0.900
**ReliefF**	34	0.957	0.953	0.960	0.988	0.913
**Symmetrical Uncertainty**	45	0.950	0.947	0.953	0.978	0.900

Table S7. Second-layer evaluation with 65 machine learning approaches.

Algorithm		Cross-validation				Independent-testing			
		Sn	Sp	Acc	MCC	Sn	Sp	Acc	MCC
Bayes	BayesNet	0.927	0.966	0.946	0.894	0.884	0.733	0.869	0.482
	DMNBtext	0.927	0.926	0.926	0.853	0.906	0.600	0.876	0.429
	NaiveBayes	0.907	0.940	0.923	0.847	0.913	0.600	0.882	0.443
	NaiveBayesLogisticRegression	0.947	0.973	0.960	0.920	0.920	0.600	0.889	0.459
	NaiveBayesSimple	0.913	0.940	0.926	0.853	0.913	0.600	0.882	0.443
	NaiveBayesUpdateable	0.907	0.940	0.923	0.847	0.913	0.600	0.882	0.443
Functions	LIBSVM	0.960	0.953	0.957	0.913	0.949	0.600	0.882	0.534
	Logistic	0.987	0.987	0.987	0.973	0.935	0.533	0.895	0.443
	MultilayerPerceptron	1.000	0.987	0.993	0.987	0.928	0.600	0.895	0.476
	RBFNetwork	0.933	0.953	0.943	0.886	0.920	0.600	0.889	0.459
	SimpleLogistic	0.980	0.980	0.980	0.960	0.928	0.600	0.895	0.476
	SMO	0.973	0.973	0.973	0.946	0.935	0.600	0.902	0.494
	SPegasos	0.987	0.993	0.990	0.980	0.920	0.600	0.889	0.459
	VotedPerceptron	0.933	0.960	0.946	0.893	0.942	0.533	0.902	0.462
Lazy	IB1	1.000	0.993	0.997	0.993	0.899	0.600	0.869	0.415
	IBk	1.000	0.993	0.997	0.993	0.899	0.600	0.869	0.415
	Kstar	1.000	0.993	0.997	0.993	0.928	0.600	0.895	0.476
	LWL	0.900	0.966	0.933	0.868	0.877	0.667	0.856	0.424
Meta	AdaBoostM1	0.953	0.993	0.973	0.947	0.884	0.667	0.863	0.436
	AttributeSelectedClassifier	0.953	0.973	0.963	0.927	0.891	0.600	0.863	0.402
	Bagging	0.960	0.960	0.960	0.920	0.891	0.600	0.863	0.402
	ClassificationViaClustering	0.867	0.913	0.890	0.780	0.862	0.733	0.850	0.446
	ClassificationViaRegression	0.980	0.987	0.983	0.967	0.928	0.600	0.895	0.476
	CVParameterSelection	1.000	0.000	0.502	0.000	1.000	0.000	0.902	0.000
	Dagging	0.927	0.960	0.943	0.887	0.928	0.600	0.895	0.476
	Decorate	0.993	0.987	0.990	0.980	0.913	0.533	0.876	0.394
	END	1.000	0.966	0.983	0.967	0.877	0.667	0.856	0.424
	FilteredClassifier	0.953	0.980	0.967	0.933	0.920	0.667	0.895	0.507
	Grading	1.000	0.000	0.502	0.000	1.000	0.000	0.902	0.000
	LogitBoost	0.980	0.987	0.983	0.967	0.891	0.733	0.876	0.495
	MultiBoostAB	0.887	0.946	0.916	0.834	0.884	0.667	0.863	0.436
	MultiClassClassifier	0.987	0.987	0.987	0.973	0.935	0.533	0.895	0.443
	MultiScheme	1.000	0.000	0.502	0.000	1.000	0.000	0.902	0.000
	OrdinalClassClassifier	1.000	0.966	0.983	0.967	0.877	0.667	0.856	0.424
	RacedIncrementalLogitBoost	1.000	0.000	0.502	0.000	1.000	0.000	0.902	0.000
	RandomCommittee	1.000	0.993	0.997	0.993	0.920	0.533	0.882	0.409
	RandomSubSpace	0.940	0.960	0.950	0.900	0.928	0.533	0.889	0.425

	**RotationForest**	1.000	0.987	0.993	0.987	0.935	0.533	0.895	0.443
	**Stacking**	1.000	0.000	0.502	0.000	1.000	0.000	0.902	0.000
	**StackingC**	1.000	0.000	0.502	0.000	1.000	0.000	0.902	0.000
	**ThresholdSelector**	0.000	1.000	0.498	0.000	0.000	1.000	0.098	0.000
	**Vote**	1.000	0.000	0.502	0.000	1.000	0.000	0.902	0.000
**Misc**	**HyperPipes**	1.000	0.711	0.856	0.744	0.942	0.467	0.895	0.409
	**VFI**	0.900	0.993	0.946	0.897	0.819	0.667	0.804	0.344
**Rules**	**ConjunctiveRule**	0.867	0.960	0.913	0.830	0.862	0.667	0.843	0.401
	**DecisionTable**	0.927	0.980	0.953	0.908	0.899	0.667	0.876	0.462
	**DTNB**	0.927	0.987	0.957	0.915	0.899	0.667	0.876	0.462
	**JRip**	0.900	0.987	0.943	0.890	0.855	0.533	0.824	0.299
	**NNge**	0.993	1.000	0.997	0.993	0.928	0.600	0.895	0.476
	**OneR**	0.887	0.940	0.913	0.827	0.884	0.600	0.856	0.389
	**PART**	0.993	0.987	0.990	0.980	0.928	0.600	0.895	0.476
	**Ridor**	0.933	0.966	0.950	0.900	0.877	0.600	0.850	0.378
	**ZeroR**	1.000	0.000	0.502	0.000	1.000	0.000	0.902	0.000
**Trees**	**ADTree**	0.987	0.993	0.990	0.980	0.891	0.600	0.863	0.402
	**BFTree**	1.000	0.980	0.990	0.980	0.906	0.533	0.869	0.379
	**DecisionStump**	0.887	0.946	0.916	0.834	0.884	0.667	0.863	0.436
	**FT**	0.993	0.980	0.987	0.973	0.957	0.600	0.922	0.557
	**J48**	1.000	0.966	0.983	0.967	0.877	0.667	0.856	0.424
	**J48graft**	1.000	0.966	0.983	0.967	0.877	0.667	0.856	0.424
	**LADTree**	0.993	0.993	0.993	0.987	0.891	0.533	0.856	0.353
	**LMT**	1.000	0.993	0.997	0.993	0.899	0.667	0.876	0.462
	**NBTree**	0.987	0.993	0.990	0.980	0.906	0.467	0.863	0.329
	**RandomForest**	0.993	1.000	0.997	0.993	0.913	0.533	0.876	0.394
	**RandomTree**	1.000	0.993	0.997	0.993	0.891	0.667	0.869	0.449
	**REPTree**	0.927	0.946	0.936	0.873	0.899	0.533	0.863	0.366
	**SimpleCart**	0.993	0.966	0.980	0.960	0.899	0.533	0.863	0.366

2 Supplementary Methods

2.1 CpG-Islands (CGIs)

$$\text{CGI\_Number} = \begin{cases} j, \ j \in \text{N} \\ 0, \text{otherwise} \end{cases} \tag{S1}$$

$$\text{CGI\_LengthRatio} = \frac{\text{length of CGI}}{\text{length of sequence}} \tag{S2}$$

$$\text{CGI\_Dis} = |\text{TLS} - \text{CGI location site}| \tag{S3}$$

$$\text{CGI\_CGRatio} = \frac{\text{CpG percent in CGI}}{\text{CGI\_Number}} \tag{S4}$$

$$\text{CGI\_OE} = \frac{\text{number of CpGs in CGI}}{(\text{number of Cs in CGI}) \times (\text{number of Gs in CGI})} \tag{S5}$$

Number coding was represented by the number of CGIs (*j*) predicted in the sequence (Equation S1). Length coding consisted of the ratio of the length of the CGIs dived by the length of the PROMOTER **or** MIDDLE sequence (Equation S2). Distance was encoded as the distance from the CpG-island to the TLS (Equation S3). The CG ratio was calculated as the ratio of CpG fragments in the CpG-island by dividing total number of CGIs (Equation S4). The OE value indicates the ratio of the number of CpGs present in the CpG-island relative to the expected value of CpG fragments and was calculated by dividing the number of CpG fragments in the sequence by the product of the number of Cs and the number of Gs in the CpG-island (Equation S5).

2.2 Kmer and RevKmer

$$\text{Kmer\_Number}_{(i)} = \begin{cases} j,\ j \in \text{N} \\ 0,\ \text{otherwise} \end{cases},\ i \in \{1, 2, \cdots, 349504\} \tag{S6}$$

$$\text{RevKmer\_Number}_{(i)} = \begin{cases} j,\ j \in \text{N} \\ 0,\ \text{otherwise} \end{cases},\ i \in \{1, 2, \cdots, 174920\} \tag{S7}$$

where i indicates kinds of nucleotide combinations and j indicates the number of matches with pattern *(i)* in a specific sequence. If there are no matches for a pattern in a specific sequence, this is coded as zero.

2.3 Nucleotide Physicochemical and Conformation Properties (DNP and TNP)

$$\text{DNP\_Value}_{(i,j)} = \frac{S(d_i)\times \text{F}_j(d_i)}{\text{sequence legnth} - 1},\ i \in \{1,2,\cdots,16\},\ j \in \{1,2,\cdots,15\},\ d_i \in \text{D},\ \text{F}_j \in F \tag{S8}$$

$$\text{TNP\_Value}_{(i,j)} = \frac{S(d_i)\times \text{F}_j(d_i)}{\text{sequence legnth} - 1},\ i \in \{1,2,\cdots,64\},\ j \in \{1,2,\cdots,12\},\ d_i \in \text{D},\ \text{F}_j \in F \tag{S9}$$

For the encoding of DNP, i indicates the kind of dinucleotide (two-nucleotide combination), and j indicates the kind of physicochemical structure of the dinucleotide. D is the collection of 16 dinucleotides (i), F is the 15 integrated dinucleotide properties (j), S(D) is the number of times that the 16 dinucleotides appear in the target sequence, F(D) is the value of the 15 properties

corresponding to 16 dinucleotides (Equation S8). For the encoding of TNP, D is the collection of 64 trinucleotides (*i*), F is the 12 trinucleotide properties (*j*), S(D) is the number of times that the 64 dinucleotides appear in the target sequence, F(D) is the value of the 12 properties corresponding to the 64 dinucleotides (Equation S9). The target sequence minus one indicates the maximum number of dinucleotide could match on sequence.

2.4 Autocorrelation (DACC and TACC)

$$D = R_1 R_2 R_3 R_4 R_5 R_6 \cdots R_L \tag{S10}$$

$$\mathrm{DACC}_{(u_1, u_2, lag)} = \sum_{i=1}^{L-lag} \frac{\left(P_{u_1}(R_i R_{i+1}) - \overline{P_{u_1}}\right)\left(P_{u_2}\left(R_{i+lag} R_{i+lag} \quad \right) - \overline{P_{u_2}}\right)}{L - lag - 1} \tag{S11}$$

$$\mathrm{TACC}_{(u_1, u_2, lag)} = \sum_{i=1}^{L-lag-2} \frac{\left(P_{u_1}(R_i R_{i+1} R_{i+2}) - \overline{P_{u_1}}\right)\left(P_{u_2}\left(R_{i+lag} R_{i+lag+1} R_{i+lag+1}\right) - \overline{P_{u_2}}\right)}{L - lag - 2} \tag{S12}$$

For a DNA sequence D with *L* nucleic acid residues, where R_1 represents the nucleic acid residue at the sequence position 1, R_2 the nucleic acid residue at position 2 and so on (Equation S10). DACC and TACC measure the correlation of the same physicochemical index between two dinucleotides or trinucleotides separated by a distance of *lag* along the sequence (Equations S11 and S12). In these equations, u_1 and u_2 are two different physicochemical indices, $P_u(R_iR_{i+1})$ and $P_u(R_iR_{i+1}R_{i+2})$ are the numerical value of the physicochemical index *u* for the dinucleotide R_iR_{i+1} or trinucleotide $R_iR_{i+1}R_{i+2}$ at position *i* and $\overline{P_u}$ is the average value for the physicochemical index value *u* along the whole sequence. The number of features represented by DACC and TACC, will be the lag value multiplied by the square of the properties number.

2.5 Logistic regression

$$\pi(\mathrm{x}) = \frac{\exp^{(1.349 - 6.592\mathrm{e}{-05}\mathrm{x})}}{1 + \exp^{(1.349 - 6.592\mathrm{e}{-05}\mathrm{x})}} \tag{S13}$$

The linear regression of distance and gene activation was expressed as $1.349 - (6.592 \times 10^{-5})x$, where *x* represents the distance variable (independent variable), *π(x)* is the dependent variable of the linear regression after the logistic conversion, representing the possibility of gene activation. The biggest difference with traditional linear regression is that the dependent variable is discrete; in addition, the resulting values range between 0 and 1.
